# Draft Genome Sequence of an Epibiotic Bacterium, Bacillus cereus, Isolated from Cyanobacterial Blooms in Lake Taihu, China

**DOI:** 10.1128/mra.00936-22

**Published:** 2023-02-13

**Authors:** Xiaoyuan Chen, Yinuo Yang, Yang Yu, Qisen Guo, Somin Hwang, Wenduo Cheng, Huansheng Cao

**Affiliations:** a Division of Natural and Applied Sciences, Duke Kunshan University, Kunshan, Jiangsu, China; University of Delaware College of Engineering

## Abstract

Here, we report the draft genome sequence of Bacillus cereus strain THSB-6-2, which was isolated from cyanobacterial blooms in Lake Taihu, China. The 5,496,658-bp genome assembly of Bacillus cereus consists of 28 contigs, with a GC content of 35% and with 5,587 protein-coding sequences and 58 RNA genes.

## ANNOUNCEMENT

Lake Taihu is one of the largest freshwater lakes in China, where cyanobacterial blooms have caused serious long-term environmental and social problems (1, 2). It has been reported that the interactions between aquatic microalgae and their symbiotic bacteria can contribute to the control of cyanobacterial blooms (3). Bacillus cereus, a widely distributed bacterial group, comprises aerobic, or facultatively anaerobic, Gram-positive bacteria with rod shapes (4, 5). B. cereus strains have been found to have lytic activities against many bloom-forming cyanobacteria, seriously damaging chlorophyll and decreasing photosynthetic activity (6, 7).

Here, we present the draft genome sequence of a Bacillus cereus strain that was one of the epibiotic bacteria isolated from surface water with cyanobacterial blooms from Lake Taihu, China (31°20′N, 120°19′E). The collected bloom water samples were filtered using filter papers with pore sizes of 30 μm, to eliminate abiotic impurities and collect cyanobacterial clumps. After filtration, free-living bacteria were removed by washing the filter paper by filtration with sterile deionized water. The retained cyanobacteria and attached epibiotic bacteria were recovered with phosphate-buffered saline (PBS) and then streaked onto BG11 plates. The streaked plates were incubated at room temperature (25°C), and no single bacterial colony was observed. The colonies of cyanobacteria with attached epibionts were picked from the plates for passage cultivation for 1 week with a 12-h light/12-h dark cycle. Cyanobacterial colonies were then streaked on plates of 90% BG11/10% LB medium with 48-h dark treatment to remove cyanobacteria and collect epibiotic bacteria. Single epibiotic bacterial colonies were picked based on morphologies and streaked on LB plates for constant passage cultivation until pure colonies were obtained. No cyanobacterial colonies were observed on the plates, and the isolated bacterial colonies were sent for sequencing.

Genomic DNA was extracted and purified using the Wizard genomic DNA purification kit (Promega, USA) according to the manufacturer's protocol. The concentration and quality parameters of the purified DNA were determined using a TBS-380 fluorometer (Turner BioSystems Inc., USA), with high-quality DNA being reserved (optical density at 260 nm [OD_260_]/OD_280_, 1.8 to 2.0; DNA amount, ≥1 μg; concentration, ≥20 ng/μL). Whole-genome sequencing was performed on the HiSeq X Ten platform (Illumina, USA); the genomic DNA was fragmented with a focused ultrasonicator (Covaris, USA), and the DNA library was prepared using the NEXTFLEX Rapid DNA-sequencing kit (PerkinElmer, USA). In total, 6,912,128 paired-end raw reads (2 × 150 bp) were produced.

All data-processing tools were used with default settings. FastQC (v0.11.9) was used to assess the quality of the raw sequencing data (8), and Trimmomatic (v0.40) was applied to trim the raw reads (9). The trimmed reads were *de novo* assembled with SPAdes (v3.13.0) (10), and the annotation of the assembled genome was obtained with the RAST online server (https://rast.nmpdr.org/) (11). The bacterial taxon was identified to be Bacillus cereus with the online Type Strain Genome Server (TYGS) (https://tygs.dsmz.de/) (12). BUSCO (v5.4.3) was used to calculate the genome completeness with the *Bacillales* database (13). The analysis produced 28 contigs, with an *N*_50_ value of 259,630 bp. The whole genome of B. cereus strain THSB-6-2 has a total size of 5,496,658 bp, with a GC content of 35%, a genome completeness of 99.8%, and genome coverage of 180×. The annotation identified 5,587 protein-coding sequences and 58 RNA genes. This work could provide the basis for further understanding of the aquatic microbial interactions involving Bacillus cereus and the potential of applying these interactions to control water blooms.

### Data availability.

The genome sequence of B. cereus was deposited in GenBank under the accession number JANIAT000000000. The raw reads were deposited in the Sequence Read Archive (SRA) under the accession number SRR20608308, with BioProject accession number PRJNA861908 and BioSample accession number SAMN29932819.
